# Changing metastatic patterns associate with dynamics of circulating tumor DNA in metastatic castration-resistant prostate cancer

**DOI:** 10.1093/oncolo/oyaf107

**Published:** 2025-05-16

**Authors:** Vincenza Conteduca, Emanuela Scarpi, Alice Rossi, Fabio Ferroni, Giorgia Gurioli, Sara Bleve, Caterina Gianni, Giuseppe Schepisi, Nicole Brighi, Cristian Lolli, Maria Concetta Cursano, Alessandra Virga, Chiara Casadei, Amelia Altavilla, Alberto Farolfi, Paola Ulivi, Domenico Barone, Federica Matteucci, Ugo De Giorgi

**Affiliations:** Unit of Medical Oncology and Biomolecular Therapy, Department of Medical and Surgical Sciences, University of Foggia, Policlinico Riuniti, 71122 Foggia, Italy; Unit of Biostatistics and Clinical Trials, IRCCS Istituto Romagnolo per lo Studio dei Tumori (IRST) “Dino Amadori,” 47014 Meldola, Italy; Radiology Unit, IRCCS Istituto Romagnolo per lo Studio dei Tumori (IRST) “Dino Amadori,” 47014 Meldola, Italy; Radiology Unit, IRCCS Istituto Romagnolo per lo Studio dei Tumori (IRST) “Dino Amadori,” 47014 Meldola, Italy; Biosciences Laboratory, IRCCS Istituto Romagnolo per lo Studio dei Tumori (IRST) “Dino Amadori,” 47014 Meldola, Italy; Department of Medical Oncology, IRCCS Istituto Romagnolo per lo Studio dei Tumori (IRST) “Dino Amadori,” 47014 Meldola, Italy; Department of Medical Oncology, IRCCS Istituto Romagnolo per lo Studio dei Tumori (IRST) “Dino Amadori,” 47014 Meldola, Italy; Department of Medical Oncology, IRCCS Istituto Romagnolo per lo Studio dei Tumori (IRST) “Dino Amadori,” 47014 Meldola, Italy; Department of Medical Oncology, IRCCS Istituto Romagnolo per lo Studio dei Tumori (IRST) “Dino Amadori,” 47014 Meldola, Italy; Department of Medical Oncology, IRCCS Istituto Romagnolo per lo Studio dei Tumori (IRST) “Dino Amadori,” 47014 Meldola, Italy; Department of Medical Oncology, IRCCS Istituto Romagnolo per lo Studio dei Tumori (IRST) “Dino Amadori,” 47014 Meldola, Italy; Biosciences Laboratory, IRCCS Istituto Romagnolo per lo Studio dei Tumori (IRST) “Dino Amadori,” 47014 Meldola, Italy; Department of Medical Oncology, IRCCS Istituto Romagnolo per lo Studio dei Tumori (IRST) “Dino Amadori,” 47014 Meldola, Italy; Department of Medical Oncology, IRCCS Istituto Romagnolo per lo Studio dei Tumori (IRST) “Dino Amadori,” 47014 Meldola, Italy; Department of Medical Oncology, IRCCS Istituto Romagnolo per lo Studio dei Tumori (IRST) “Dino Amadori,” 47014 Meldola, Italy; Biosciences Laboratory, IRCCS Istituto Romagnolo per lo Studio dei Tumori (IRST) “Dino Amadori,” 47014 Meldola, Italy; Radiology Unit, IRCCS Istituto Romagnolo per lo Studio dei Tumori (IRST) “Dino Amadori,” 47014 Meldola, Italy; Nuclear Medicine Operative Unit, IRCCS Istituto Scientifico Romagnolo per lo Studio dei Tumori (IRST) “Dino Amadori,” 47014 Meldola, Italy; Department of Medical Oncology, IRCCS Istituto Romagnolo per lo Studio dei Tumori (IRST) “Dino Amadori,” 47014 Meldola, Italy

**Keywords:** ctDNA, metastatic pattern, bone metastasis, mCRPC, androgen receptor signaling inhibitors

## Abstract

**Background:**

Circulating tumor DNA (ctDNA) acts as an early biomarker of the efficacy of androgen receptor signaling inhibitor (ARSI) therapy. In this study, we aimed to reveal if ctDNA can supplement imaging to better predict metastasis burden and radiographic progression disease (PD) in metastatic castration-resistant prostate cancer (mCRPC).

**Methods:**

Targeted next-generation sequencing was performed to assess ctDNA fraction. Radiographic evidence was documented by conventional imaging according to Prostate Cancer Working Group 3 criteria.

**Results:**

We prospectively collected plasma samples from 112 mCRPC with bone (*n* = 77), lymph nodal (*n* = 31), and visceral (*n* = 4) metastases. Only bone metastatic patterns were significantly associated with median ctDNA at baseline, during treatment and at PD (*P* <.0001). At first radiographic restaging, 24 (31.2%) men with a progressive worsening of bone disease had early ctDNA rise with a % ctDNA variation of 150.6% (interquartile range [IQR] = 104.9-210.7] compared with 11.1% (IQR = 0-36.6), *P* <.0001, in men with no change in bone disease. Univariate analysis showed that early ctDNA rise was significantly associated with progression free/overall survival (PFS/OS). In multivariable analysis including ctDNA change from baseline to 3-month treatment, variation of bone metastatic patterns (from oligometastatic to polymetastatic and/or to widespread disease), presence of visceral metastasis, age, PSA, performance status and prior docetaxel therapy, the transition from low- to high-ctDNA within 3 months of starting ARSI therapy was a significant predictor of OS (HR = 2.50, 90% CI, 1.06-5.88, *P* =.035) and persistent high level of ctDNA was a predictor of PFS (HR = 2.53, 95% CI, 1.10-5.81, *P* =.028). Metastatic involvement demonstrated that the transition from bone polymetastatic to widespread disease and the presence of visceral metastases were both associated with worse OS (HR = 2.43, 95% CI, 1.10-5.35, *P* =.028, and HR = 3.40, 95% CI, 1.50-7.66, *P* =.003, respectively). Prior therapy with docetaxel represented an independent predictor of both PFS and OS (HR = 2.47, 95% CI, 1.40-4.35, *P* =.002, and HR = 1.78, 95% CI, 1.00-3.15, *P* =.049, respectively).

**Conclusions:**

Early ctDNA variation might reflect changes in metastatic burden and, likely, in bone metastatic patterns on ARSI therapy allowing to track pattern of disease progression and to predict outcome.

**Implications for Practice**

This study showed that ctDNA analysis can supplement imaging to better predict metastasis burden in metastatic castration-resistant prostate cancer (mCRPC) patients receiving androgen receptor signaling inhibitors. In particular, dynamic changes in ctDNA may reflect changes in bone metastatic pattern on therapy and early increase of ctDNA fraction is associated with poor survival in mCRPC. These findings suggested that ctDNA could be complementary to imaging to early predict outcome in mCRPC patients.

## Introduction

Prostate cancer is the most frequently diagnosed cancer and the second leading cause of cancer death in men.^1^ Patients with metastatic hormone-sensitive prostate cancer (mHSPC) usually respond to androgen deprivation therapy but invariably relapse with metastatic castration-resistant prostate cancer (mCRPC) within a median of 18-24 months leading to emergence of a lethal phenotype.^2^

In the castration-resistant setting, 70%-90% of patients have radiographically detectable bone involvement, and 40% will have soft tissue metastases (lymph node, local recurrence, and visceral lesions), leading to significantly increase the disease-specific morbidity and mortality.^3,4^ Therefore, understanding the potential organ tropism and progression disease (PD) of prostate tumor could be critical for an effective personalized follow-up strategy.

There are currently no diagnostic tools with the ability to predict the pattern of recurrence and promote monitoring and design of organ-specific interventions. The Prostate Cancer Clinical Trials Working Group 3 (PCWG3) recommendations^5^ highlighted the importance of recognizing the biology of mCRPC, actionable molecular aberrations, and tumor heterogeneity to improve the baseline patient assessment and the evaluation of PD in association with modern and conventional imaging techniques.

Liquid biopsy is progressively used as a biomarker of disease status in multiple types of cancers, including prostate cancer, providing real-time information through the detection of circulating tumor DNA (ctDNA), circulating tumor cells, or exosomes for better prognostication and genomic characterization.^6-8^

The role of plasma DNA dynamics has been recently demonstrated as an early predictor of therapy efficacy for mCRPC.^9-13^ In addition, there are some studies^13-16^ that have also defined the clinical validity of ctDNA to effectively identify the pattern of PD in advanced prostate cancer patients treated with other types of treatment for mCRPC such as chemotherapy or PARP inhibitors.

Here, we aimed to reveal if ctDNA analysis can help to predict metastatic burden and tumor dynamics in mCRPC patients.

## Methods

### Study design

Our biomarker study (REC 2192/2013) was approved by the institutional ethics committee of the IRCCS Istituto Romagnolo per lo Studio dei Tumori (IRST) “Dino Amadori,” 47014 Meldola, Italy, and was carried out in accordance with the requirements of the International Conference on Harmonization E6 for Good Clinical Practice as laid down in the Helsinki Declaration. All patients included provided written informed consent. These participants were required to have histologically confirmed prostate adenocarcinoma without neuroendocrine differentiation, a metastatic disease with only one metastasis site, PD despite “castration levels” of serum testosterone (<50 ng/dL), on-going luteinizing hormone-releasing hormone analogue therapy or prior surgical castration. Patients received a treatment with androgen receptor signaling inhibitor (ARSI) for mCRPC: abiraterone 1 g once a day and prednisone 5 mg twice daily or enzalutamide 160 mg once daily as first-line or second-line therapy. Androgen receptor signaling inhibitors were administered continuously until evidence of PD or unacceptable toxicity. We excluded patients receiving prior ARSI therapy for mHSPC and mCRPC to avoid cross-resistance between second-generation hormonal agents.

Radiographic evaluation consisted of the use of computed tomography (CT) and bone scan at the time of screening and every 12 weeks on treatment. The use of positron emission tomography (PET)/CT with 18F-fluorocholine or prostate-specific membrane antigen (PSMA) ligand was optional. Serum prostate-specific antigen (PSA) was assessed within 3 days of starting therapy and monthly thereafter.

### Circulating tumor DNA fraction analysis

Blood samples were processed into plasma within 3 hours of collection. Circulating DNA was extracted from 1 to 2 mL of plasma from each patient using the QIAamp Circulating Nucleic Acid Kit (Qiagen) and quantified using the high-sensitivity Quant-iT PicoGreen double-stranded DNA Assay Kit (Invitrogen).^17^

In plasma and patient-matched germline DNA, targeted next-generation sequencing (NGS) was assessed by using the PGM Ion Torrent with a 316 or 318 Chip to account for 1000× expected coverage per target. We estimated the tumor content for each blood sample from study patients by performing a computational analysis previously described.^18^ The ctDNA fraction for each blood sample both at baseline and during treatment was assessed using the CLONET computational tool evaluating genomic deletions. The accuracy of this method to estimate ctDNA fraction was recently confirmed using targeted-methylome-NGS for 5.5 million pan-genome CpG sites where the main driver of methylome variance in plasma from mCRPC had a very strong correlation with ctDNA amount determined by CLONET.^19^

### Statistical analysis

Primary endpoint of the study was to determine if ctDNA associates with metastatic pattern at different time points (baseline, on treatment, at PD). Secondary endpoints were to evaluate the role of early ctDNA variation in monitoring the response to treatment with abiraterone or enzalutamide. The radiographic and biochemical responses were defined according to PCWG3 criteria.^5^

Radiographic progression-free survival (PFS) was calculated from the beginning of ARSI to the date of PD or death, whichever occurs first, or last tumor assessment. Overall survival (OS) was calculated from the first day of treatment until death or last follow-up.

Survival curves were estimated by the Kaplan-Meier method and were compared using the log-rank test. Cox regression models were used to explore potential factors which could predict PFS and OS and to estimate hazard ratios (HRs) and their 95% CI. All *P*-values were 2-sided and a *P* <.05 was considered as statistically significant. Statistical analyses were performed with SAS 9.4 software (SAS Institute, Cary, NC, USA).

The impact of change of ctDNA and metastatic pattern at 3 months on survival outcomes was assessed by landmark analyses. Patients with early progressive disease/death before the landmark times were excluded. For these analyses, PFS and OS times were measured from the landmark times to these survival outcomes.

## Results

### Patient characteristics

After appropriate consent, a total of 326 plasma specimens were collected from 112 patients with mCRPC between June 2013 and January 2018, at the time of initiation of a new line of treatment (baseline specimen), during treatment, and at PD. Supplementary Table 1 shows the number of plasma samples in each time point. The median age of the patients was 74 [IQR 69-79 years]. In total, 67 (59.8%) patients had a Gleason score of 8 and 103 (92.0%) had Eastern Cooperative Oncology Group (ECOG) performance status (PS) 0-1. Baseline PSA value was 36.2 ng/mL (IQR 11.9-154.5). Sixty-four (57.1%) patients received abiraterone and 48 (42.9%) enzalutamide. Most patients (*n* = 68, 60.7%) had prior therapy with docetaxel.

All (100%) patients had metastatic disease, whose 26 (23.2%) had presented with metastases at the time of their initial prostate cancer diagnosis. For patients who had initially presented with clinically localized prostate cancer, 55 (49.1%) underwent prostatectomy and 31 (27.7%) received radiotherapy.

In the present study, we included patients with only one metastasis site consisting of bone, lymph nodal, and visceral involvement in 77 (68.7%), 31 (27.7%), and 4 (3.6% [3 liver, 1 lung]) cases, respectively, to avoid bias in the interpretation of results. Clinical features of mCRPC patients are described in Table 1.

**Table 1.** Patient characteristics.

	*N* (%)
**Age** (years), median value (IQR)	74 (69-79)
**Gleason score**	
6-7	45 (40.2)
≥8	67 (59.8)
**ECOG PS**	
0-1	103 (92.0)
2	9 (8.0)
**Prostatectomy**	
No	57 (50.9)
Yes	55 (49.1)
**Radiotherapy**	
No	81 (72.3)
Yes	31 (27.7)
**Type of therapy**	
Abiraterone	64 (57.1)
Enzalutamide	48 (42.9)
**Prior docetaxel**	
No	44 (39.3)
Yes	68 (60.7)
**Number of prior therapies**	
0-1	74 (66.1)
≥2	38 (33.9)
**Sites of metastasis**	
Bone	77 (68.7)
Lymph nodes	31 (27.7)
Visceral	4 (3.6)
**PSA**, median value (IQR), ng/dL	36.2 (11.3-154.5)
**ctDNA fraction,** median value (IQR)	0.180 (0.11-0.38)

Abbreviations. ctDNA, circulating tumor DNA; ECOG, Eastern Cooperative Oncology Group; *N*, number; PS, performance status; PSA, prostate-specific antigen.

### Association of ctDNA and metastatic pattern during treatment

Median ctDNA fraction was 0.180 (IQR 0.11-0.38). Patients were divided into 2 groups: “high ctDNA” (men with ctDNA ≥ 0.180) and “low ctDNA” (men with ctDNA < 0.180), as reported by previous evidence that used median value of ctDNA fraction as threshold for the correlation of plasma DNA with outcomes and other clinical features in mCRPC patients.^18^ Here, we examined the correlation of ctDNA fraction with sites of metastasis, as determined through conventional imaging.

Bone disease, documented by conventional imaging according to PCWG3 criteria,^5^ was classified on the basis of metastasis number (≤5, 6-19, and ≥20) in oligometastatic (O), polymetastatic (D), and widespread (S), respectively.

Patients with high ctDNA were significantly associated with higher number of bone metastases at baseline (*P* =.0003), first radiographic evaluation (*P* =.0003), and at PD (*P* =.0007). No association was reported between ctDNA fraction and the presence of pathological tissue and/or pathological fracture (Table 2).

**Table 2.** Association of ctDNA and metastatic pattern in mCRPC during treatment.

	ctDNA low	ctDNA high	
	*N* (%)	*N* (%)	*P*
**Bone metastases**			
*Baseline*			
* Bone extent*			
Oligometastatic (≤5 lesions) (O)	24 (61.6)	10 (26.3)	
Polymetastatic (between 6 and 19 lesions) (D)	10 (25.6)	7 (18.4)	
Widespread (≥20 lesions) (S)	5 (12.8)	21 (55.3)	.0003
Presence of pathological tissue	2 (2.7)	5 (6.8)	.442
Presence of pathological fracture	1 (1.4)	4 (5.4)	.366
*First radiological assessment*			
* Bone extent*			
Oligometastatic (≤5 lesions) (O)	20 (50.0)	4 (10.8)	
Polymetastatic (between 6 and 19 lesions) (D)	13 (32.5)	12 (32.4)	
Widespread (≥20 lesions) (S)	7 (17.5)	21 (56.8)	.0003
Presence of pathological tissue	0	0	-
Presence of pathological fracture	1 (1.4)	3 (4.1)	.620
*Final (PD) assessment*			
* Bone extent*			
Oligometastatic (≤5 lesions) (O)	15 (35.7)	2 (5.7)	
Polymetastatic (between 6 and 19 lesions) (D)	13 (31.0)	9 (25.7)	
Widespread (≥20 lesions) (S)	14 (33.3)	24 (68.6)	.0007
Presence of pathological tissue	4 (5.4)	5 (6.8)	.731
Presence of pathological fracture	2 (2.7)	2 (2.7)	1.000
**Lymph nodal metastases**			
*Baseline*			
Lymph nodal site			
Thoracic (T)	1 (6.2)	0	
Abdominal (A)	12 (75.0)	11 (73.3)	
Both (TA)	3 (18.8)	4 (26.7)	.561
Number of lymph node metastases			
<5	9 (56.3)	7 (46.7)	
≥5	7 (43.7)	8 (53.3)	.594
Max size (cm) median value (IQR)	20 (16-32)	23 (17-34)	.297
*First radiological assessment*			
Lymph nodal site			
Thoracic (T)	1 (8.3)	0	
Abdominal (A)	8 (66.7)	6 (66.7)	
Both (TA)	3 (25.0)	3 (33.3)	.646
Number of lymph node metastases			
<5	9 (75.0)	6 (66.7)	
≥5	3 (25.0)	3 (33.3)	.676
Max size (cm) median value (IQR)	21 (13-30)	22 (14-26)	.805
*Final (PD) assessment*			
Lymph nodal site			
Thoracic (T)	1 (6.2)	0	
Abdominal (A)	12 (75.0)	11 (73.3)	
Both (TA)	3 (18.8)	4 (26.7)	.561
Number of lymph node metastases			
<5	10 (52.6)	10 (62.5)	
≥5	9 (47.4)	6 (37.5)	.557
Max size (cm) median value (IQR)	23 (19-32)	23 (18-39)	.814

Abbreviations. ctDNA, circulating tumor DNA; *N*, number; PD, progressive disease.

Conversely, we observed no correlation between the level of ctDNA fraction and the site (thoracic and/or abdominal), the number, and the size of lymph nodal metastases at different time points of ARSI treatment (Table 2).

In addition, we also reported no association between the size of prostate in patients who had not undergone prostatectomy and/or with local recurrence during ARSI treatment and ctDNA levels (Supplementary Table 2).

We did not find any correlation between ctDNA variation and visceral metastasis because of low number of patients with visceral involvement (*n* = 4).

Lastly, based on prior evidence of *AR* copy number as predictive biomarker in ARSI-treated mCRPC patients,^6,8,16,17^ we aimed to perform an exploratory analysis to associate *AR* status detected in ctDNA and metastatic pattern. In this exploratory analysis of plasma *AR* and metastatic patterns, we have considered only patients with a threshold of 0.075 as the lower ctDNA amenable to accurate estimation of absolute AR copy number, as previously demonstrated.^16^ We identified no correlation between plasma *AR* gain and higher number of metastases during longitudinal monitoring of mCRPC patients (Supplementary Table 3). Moreover, *AR*-gained patients who had not undergone prostatectomy and/or with local recurrence did not present with major prostatic involvement (Supplementary Table 4).

### Association between early variation in ctDNA fraction and pattern of bone metastases

Our data suggested a meaningful utility of ctDNA as biomarkers for longitudinal monitoring of mCRPC patients with bone metastasis. For this reason, we assessed early ctDNA changes from baseline to 3-month ARSI treatment to aid to establish therapy efficacy for bone metastases in mCRPC patients. We examined the correlation between ctDNA fraction and disease status at first restaging in 77 patients with only bone metastases and who had completed at least 3 months of ARSI treatment according to PCWG3 criteria.

Bone metastatic patterns were significantly associated with median ctDNA levels before and during ARSI treatment (*P* =.0003 and *P* =.0003, respectively) (Supplementary Table 5). Supplementary Figure 1 represented the values of ctDNA at baseline and at 3-month therapy from each patient.

**Figure 1.**
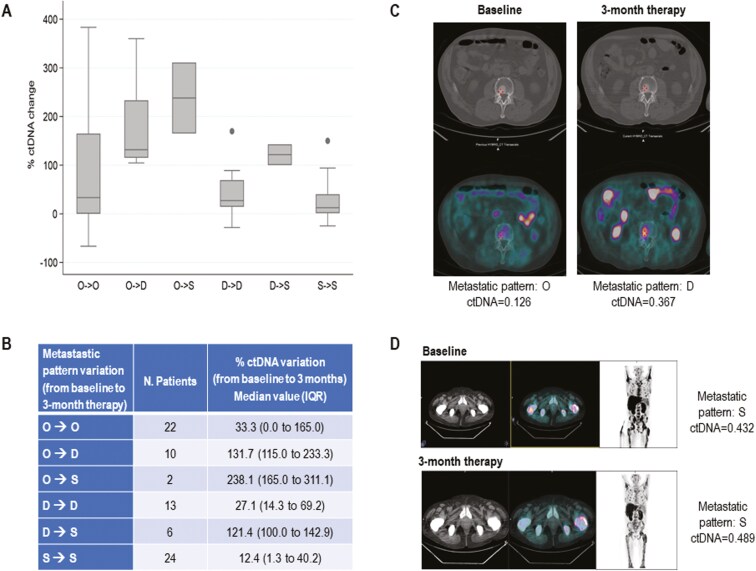
Association between variation in ctDNA fraction and pattern of bone metastases. (A) and (B) The percentage change in ctDNA fraction and the transition of bone metastatic pattern from baseline to 3-month treatment. (C) Exemplar case of mCRPC patient receiving abiraterone with transition from bone oligometastatic to bone diffuse disease with an increase of ctDNA fraction. (D) Exemplar case of mCRPC man treated with enzalutamide showing a concomitant stable widespread (S) pattern and ctDNA value at baseline and 3-month treatment. Abbreviations. ctDNA, circulating tumor DNA; O, oligometastatic (≤5 lesions); D, polymetastatic (between 6 and 19 lesions); S, widespread (≥20 lesions).

We observed a significant correlation between the percentage change in ctDNA fraction and the transition of bone metastatic pattern from baseline to 3-month treatment (Figure 1A and B). Specifically, at first radiographic evaluation, we demonstrated an early ctDNA rise, defined as an increase of the percentage of ctDNA variation from baseline to 3-month therapy, in 24 men with an overall progressive worsening of bone metastatic disease, whose 18 developed a rise of the number of bone metastases and 6 had stable number but increased size of bone metastases. At first restaging, 18 patients presented a worsening transition in bone metastatic pattern with a ctDNA variation of 150.6% (IQR 104.9-210.7) compared with 11.1% (IQR 0-36.6), *P* <.0001, in men with no change in bone disease.

Figure 1C and D showed the potential impact of early ctDNA changes in establishing tumor burden in 2 exemplar cases of mCRPC with bone metastases receiving abiraterone and enzalutamide, respectively. The first case (Figure 1C) was characterized by a transition from bone oligometastatic to bone diffuse disease with the percentage of ctDNA variation more than doubled, whereas the second case (Figure 1D) showed a concomitant stable widespread (S) pattern and ctDNA value at baseline and 3-month treatment.

### Association between early ctDNA variation and clinical outcome

Based on changes in ctDNA fraction from baseline to 3-month treatment, we considered different patient groups (Low-Low, Low-High, High-High, and High-Low) for Kaplan-Meier survival analysis. From this analysis, we excluded the category “High-Low” as it included only 4 patients. We showed that variation of ctDNA from baseline to 3-month treatment was significantly associated with worse PFS (HR = 2.06, 95% CI, 1.30-3.27, *P* =.002) and OS (HR = 1.82, 95% CI, 1.15-2.88, *P* =.010) (Supplementary Table 6).

**Figure 2.**
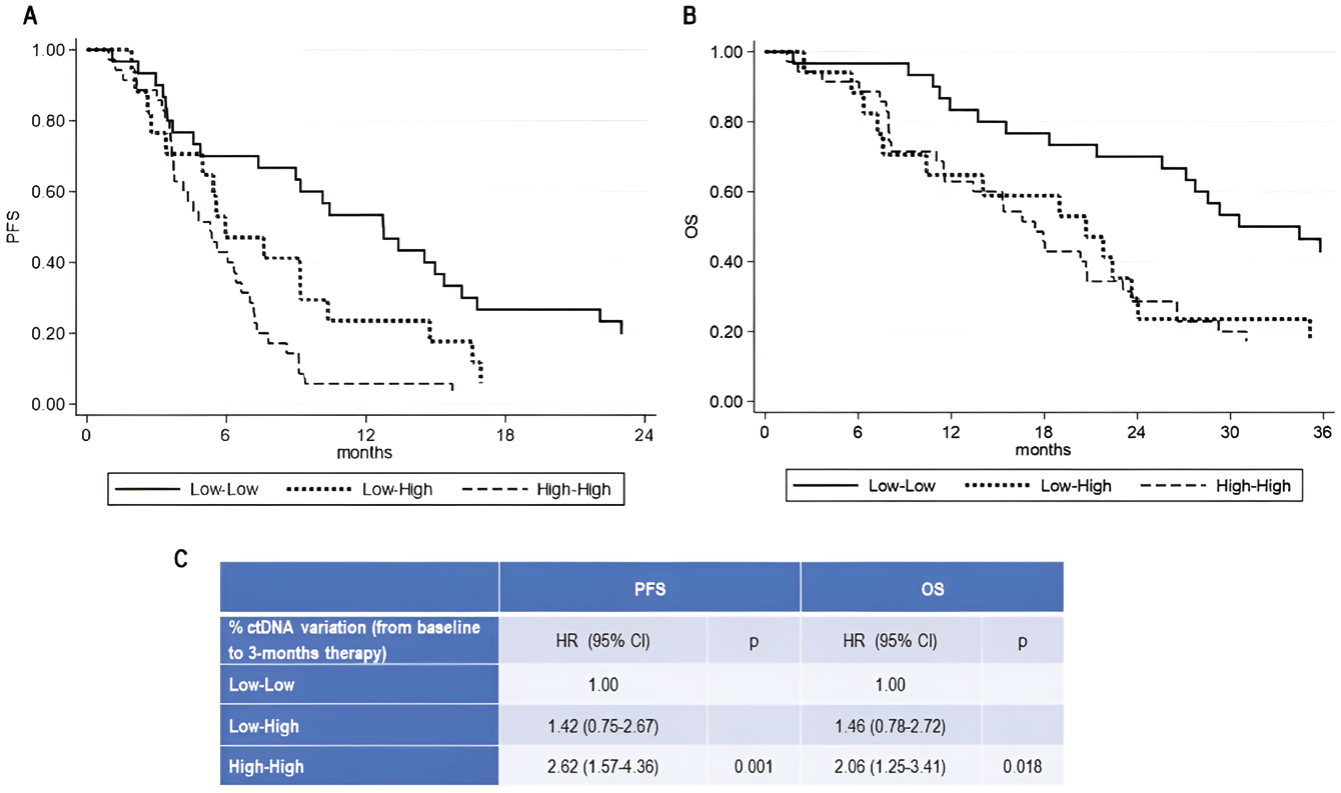
Association between early ctDNA variation and clinical outcome. (A) Progression-free survival (PFS) and (B) overall survival (OS) according to variation in ctDNA fraction from baseline to 3-month treatment (Low-Low, Low-High, and High-High); (C) Univariate analysis of PFS and OS as a function of ctDNA change.

In univariate analysis, “Low-Low” group with no change in ctDNA was characterized by a significant longer PFS and OS (*P* =.001 and *P* =.018, respectively) compared with other groups (Figure 2).

In multivariable analysis, we included factors associated with PFS and/or OS in the univariate analysis such as ctDNA variation dichotomized into high-ctDNA and low-ctDNA subgroups, 4 changing bone metastatic patterns: (1) O 27 →O, (2) D →D/D →S, (3) S →S, and (4) O →D/O →S, (presence of visceral metastasis, age, performance status, PSA value, and prior therapy with docetaxel) (Supplementary Table 7). ctDNA change at 3-month treatment showed that the transition from low- to high-ctDNA was a significant predictor of OS (HR 2.50, 90% CI, 1.06-5.88, *P* =.035) and persistent high level of ctDNA was a predictor of PFS (HR 2.53, 95% CI, 1.10-5.81, *P* =.028). Metastatic involvement demonstrated that the transition from bone polymetastatic (D) to widespread (S) disease and the presence of visceral metastasis were associated with worse OS (HR 2.43, 95% CI, 1.10-5.35, *P* =.028, and HR 3.40, 95% CI, 1.50-7.66, *P* =.003, respectively). Prior therapy with docetaxel represented independent predictor of PFS and OS (HR 2.47, 95% CI, 1.40-4.35, *P* =.002, and HR 1.78, 95% CI, 1.00-3.15, *P* =.049, respectively) (Table 3).

**Table 3.** Multivariate analysis of PFS and OS.

	PFS		OS	
	HR (95% CI)	*P*	HR (95% CI)	*P*
**% ctDNA** (from baseline to 3 months)				
Low-Low	1.00		1.00	
Low-High	1.94 (0.89-4.26)	.098	2.50 (1.06-5.88)	.035
High-High	2.53 (1.10-5.81)	.028	1.42 (0.59-3.42)	.434
**Metastatic pattern variation** (from baseline to 3 months)				
O → O	1.00		1.00	
D → D / D → S	1.19 (0.57-2.50)	.636	2.43 (1.10-5.35)	.028
S → S	0.60 (0.27-1.36)	.223	1.45 (0.61-3.41)	.399
O → D / O → S	1.15 (0.45-2.96)	.772	1.85 (0.66-5.18)	.238
**Visceral metastasis**				
No	1.00		1.00	
Yes	1.56 (0.74-3.27)	.241	3.40 (1.50-7.66)	.003
**ECOG PS**				
0-1	1.00		1.00	
2	0.68 (0.18-2.53)	.564	1.06 (0.28-4.02)	.926
**Age** (continuous variable)	1.01 (0.98-1.05)	.538	1.04 (1.00-1.07)	.053
**PSA baseline**				
<32.73 (median value)	1.00		1.00	
≥32.73	1.05 (0.58-1.91)	.868	1.10 (0.58-2.09)	.767
**Prior docetaxel**				
No	1.00		1.00	
Yes	2.47 (1.40-4.35)	.002	1.78 (1.00-3.15)	.049

Abbreviations. ctDNA, circulating tumor DNA; D, polymetastatic disease (between 6 and 19 lesions); ECOG, Eastern Cooperative Oncology Group; HR, hazard ratio; *N*, number; O, oligometastatic disease (≤5 lesions); PFS, progression-free survival; PS, performance status; PSA, prostate specific antigen; S, widespread disease (≥20 lesions).

## Discussion

The present study analyzed a cohort of mCRPC patients characterized by serial evaluation of ctDNA fraction with the aim of capturing the real-time biological features of the disease according to different sites of metastasis.

The results showed that elevated ctDNA levels were associated with higher metastatic burden and, in particular, with increased number of bone metastases. The early variation of ctDNA from baseline to 3-month treatment can predict a transition from oligometastatic to diffuse and/or widespread disease. In addition, changes in ctDNA fraction during treatment also provided prognostic information with the potential for guiding clinical management.

Clinical applicability of ctDNA testing for prognosis has been widely demonstrated in cancer, but its role as a surrogate marker of disease burden and as a monitoring tool represents one of the most driving interesting challenges for clinical utility. This approach could potentially help in tracing the heterogeneity pattern to duly tailor individual treatment, especially in the current context of a growing number of novel therapeutic strategies for advanced prostate cancer. Recent study of deep whole-genome sequencing of serial plasma and synchronous metastases in patients with aggressive prostate cancer showed genomic drivers of acquired treatment resistance.^20^ In light of this, our study confirmed the clinical utility of plasma DNA analysis as a combined approach involving both qualitative and quantitative assessment of ctDNA. Interestingly, Kohli et al.^21^ showed the distribution of ctDNA fraction across metastatic prostate cancer groups with significant differences in yield and molecular profiling and a combined analysis of ctDNA fraction and other prognostic factors, such as metastatic volume, providing a better prediction of therapeutic resistance. Moreover, the integration of liquid and tissue comprehensive genomic profiling has been demonstrated as 2 complementary tools to aid treatment choices for prostate cancer patients.^18,22,23^

Ability to track the pattern of bone progression thanks to the use of longitudinal ctDNA assessment may aid in the clinical practice as a majority of metastatic lesions are in bone but bone biopsy yield is extremely variable. Sclerotic bone lesions have been notoriously challenging to biopsy because the amount of tissue obtained is scant and requires decalcification, and often is insufficient for extensive molecular analyses.^24^ A previous study demonstrated that only 25.5% of 184 patients with metastatic prostate cancer had a positive bone biopsy.^25^ Recently, the success rates for image-guided bone biopsies in specialized academic centers have significantly improved.^26-28^ However, the broader application of bone biopsy protocols across the clinical community is still challenged by a lack of standardized protocols.

Radiographic monitoring of tumor can have some limitations which could be overcome thanks to the estimation of ctDNA dynamics. First, the sensitivity and specificity of CT and bone scan imaging can be sometimes suboptimal for the accurate evaluation of patients with bone metastasis due to the poor characteristics of conventional bone imaging.^29^ In addition, there is the phenomenon of bone flare, characterized by an incidence ranging from 10% to 50%, leading to the apparent emergence of new or more prominent bone lesions that can be misinterpreted as true progression.^30^ Third, while new radionuclide imaging techniques such as PSMA PET/CT may address the limitations of conventional imaging, the current use of PET/CT as a treatment evaluation tool is lacking.^31^

In the last years, the growing armamentarium of therapeutic options for different settings of prostate cancer emphasizes even more the use of ctDNA in the overall management of prostate cancer patients. We demonstrated that the value of ctDNA before and after treatment had important prognostic implications, consistent with literature.^12-17^ Recently, Tolmeijer et al.^11^ showed that ctDNA assessment at baseline and 4 weeks after starting therapy is associated with response durability to first-line ARSI. Baseline ctDNA was detected (≥1% ctDNA) in 59% of patients and persistent ctDNA detection 4 weeks after treatment initiation was associated with 4.8 times shorter PFS and 5.5 times shorter OS compared with patients with undetected ctDNA at baseline and 4 weeks. In contrast, subjects who convert their ctDNA from detected to undetected within 4 weeks of treatment have a survival rate similar to those with undetected ctDNA at both timepoints. In our paper, we had only 3 patients with undetectable DNA (2 baseline, 1 on treatment) perhaps due to the fact that our patients were more heavily treated (33.9% of men received ≥ 2 prior therapeutic lines) than those chemotherapy-naïve included in Tolmeijer’s study. Recently, Sweeney et al.^32^ confirmed this evidence in a phase III study of 494 enzalutamide with or without atezolizumab after abiraterone showing that baseline ctDNA level and variations in ctDNA fraction from baseline to 3-month therapy were able to identify patients more likely to manifest survival benefit from enzalutamide.

In the last years, the better characterization of different clinical settings of prostate cancer led to a more personalized approach to prostate tumor patients. Indeed, the treatment of oligometastatic prostate cancer represents a new aspect in the multimodal treatment concept with radiotherapy on the primary tumor and metastasis-directed therapy; however, little is known regarding the utility of biomarkers to guide treatment for these patients. In this context, a multimodal approach combining ctDNA estimation and imaging might aid to better identify the oligometastatic disease. Recently, Deek et al.^33^ revealed that oligometastatic HSPC patients harboring high-risk mutational signature, defined as pathogenic somatic mutations within ATM, BRCA1/2, Rb1, and/or TP53, may help risk stratify treatment outcomes after metastasis-directed therapy, underlining the importance to define novel therapeutic paradigms thanks to the role of genetic biomarkers even within this population. In addition, aberrations in DNA defect repair (DDR) genes detected in both tissue and liquid biopsy from 115 mCRPC patients were explored in association with the presence of bone metastases. Specifically, a significant association between bone metastasis volume and DDR alterations (*P* =.0325) was reported, whereas no correlation was observed with bone-related efficacy endpoints.^34^ In addition, a recent preclinical study^35^ carried on in both PC-3 and LNCaP-19 cells showed that some DNA repair genes (BRCA1, BRCA2, PALB2, BRIP1, and RAD51C) were upregulated by osteoclasts and that 2 of these (BRCA1 and PALB2) were shown to be affected also on the protein level. The reduced DNA damage, as demonstrated by lower γ-H2AX levels, in osteoclast-stimulated CRPC cells after exposure to UV-light revealed that osteoclasts could influence the response to DNA damage in CRPC cells. Thus, this in vitro evidence confirmed potential relationship between bone metastasis and different molecular pathways, including AR and DDR leading to potential combining therapeutic strategies to overcome mechanisms of resistance and to improve sensitivity to PARP inhibitors and/or hormonal drugs and/or chemotherapy.

Moreover, ctDNA might be also a predictive biomarker for bone-targeting therapeutic agents such as radium-223 due to its potential of bone disease monitoring. Indeed, in a recent phase I trial of 30 mCRPC patients receiving radium-223 plus niraparib, Quinn et al.^36^ explored the presence of mutations in ctDNA as well as gene expression variations detected in whole blood samples as potential predictive factors, even they did not perform a quantitative assessment of ctDNA.

The current work represents a secondary analysis of one of our biomarker studies including also the relationship between plasma tumor DNA and PSA kinetics in which we previously demonstrated that high ctDNA levels correlated not only with radiographic progression but also significantly with PSA kinetics in mCRPC men treated with abiraterone or enzalutamide.^37^ This further underscores the utility to perform randomized trials to demonstrate that the longitudinal assessment of multiple noninvasive factors, such as PSA and ctDNA estimation, could be complementary to imaging before clinical decisions are made. Moreover, the current work confirmed that ctDNA and the presence of visceral metastasis, mainly (75%) represented by liver metastases, were significantly associated with OS in mCRPC,^38,39^ underlining the strong correlation between ctDNA fraction and the extent of disease. In light of these findings, serial ctDNA monitoring could provide a window of opportunity for therapy intensification in patients with inadequate tumor control while sparing additional toxicity in case of a deep and durable response to treatment.

However, there are several limitations to the present study. First, the small sample size so that a larger validation is needed. Second, the clinical heterogeneity of mCRPC, especially characterized by highly variable number of previous therapies, which could impair plasma *AR* status and clinical outcome.^40^ Third, the lack of next-generation imaging such as PSMA PET/CT in most enrolled patients could have led to a better definition of tumor burden. Fourth, beyond comprehensive assessment of bone involvement and plasma DNA status, other metastatic sites and genomic alterations have not been studied in such depth. Lastly, in some cases, there could be ctDNA changes within biological/technical variability. Nevertheless, this hypothesis-generating research provides a framework to investigate such questions in the future.

## Conclusions

Metastatic disease is often difficult to accurately measure and assess radiologically. Consequently, the identification of complementary tools able to better define radiologic progression in mCRPC patients is highly recommended. In the present study, we suggested that rising in ctDNA fraction under treatment pressure, regardless of type and treatment line, can be predictive of response to ARSI therapy. In particular, early ctDNA variation might reflect changes in tumor metastatic burden and, likely, with bone metastatic pattern on ARSI allowing to track pattern of disease progression and predict outcome. Additional research is needed to evaluate the clinical utility of personalized treatment decision-making guided by dynamic changes in ctDNA in patients with mCRPC.

## Supplementary Material

oyaf107_suppl_Supplementary_Tables_1-7_Figures_1

## Data Availability

The datasets generated during and/or analyzed during the current study are available from the corresponding author on reasonable request.
