# Supplementary material for: Changing metastatic patterns associate with dynamics of circulating tumor DNA in metastatic castration-resistant prostate cancer
Source: Oncologist. 2025 May 16;30(5):oyaf107. doi: 10.1093/oncolo/oyaf107 (PMC12082820; doi:10.1093/oncolo/oyaf107)

**Supplementary Online Material**

**Supplementary Table 1.** Number (N) of plasma samples at different time points…………….……………….….2

**Supplementary Table 2.** Association of ctDNA and prostatic involvement in mCRPC…………………….….….3

**Supplementary Table 3.** Association of plasma *AR* copy number and metastatic pattern in mCRPC………....4

**Supplementary Table 4.** Association of plasma *AR* copy number and prostatic involvement in mCRPC…,,….6

**Supplementary Table 5.** Association of ctDNA fraction and metastatic pattern at baseline and 3-month therapy..………………………………………………………………………………………………………….………...7

**Supplementary Table 6.** Univariate analysis of PFS and OS as a function of ctDNA change (from baseline to 3-month therapy)……………………………………………………………………………………… ….…………,…….8

**Supplementary Table 7.** Univariate analysis of PFS and OS……………...……………………………….............9

**Supplementary Figure 1.** Variation in ctDNA fraction from baseline to 3-month therapy………………...........10

**Supplementary Table 1. Number (N) of plasma samples at different time points**

| **Number of plasma samples at different time points** | **Bone**  **metastasis**  **(N=77)** | **Lymph node metastasis**  **(N=31)** | **Visceral metastasis**  **(N=4)*** | **Total**  **(N=112)** |
| --- | --- | --- | --- | --- |
| Baseline | 77 | 31 | 4 | 112 |
| 3-month therapy | 77 | 21** | 4 | 102 |
| Progression disease | 77 | 31 | 4 | 112 |

* We did not perform any statistical analysis in patients with visceral metastasis because of too small sample size

** In patients with lymph nodal metastasis, 10 plasma samples at 3-month therapy were missed

**Supplementary Table 2. Association of ctDNA and prostatic involvement in mCRPC**

|  | **ctDNA low** | **ctDNA high** |  |
| --- | --- | --- | --- |
|  | **N (%)** | **N (%)** | **p** |
| **Primary tumour** |  |  |  |
| *Baseline* |  |  |  |
| Median size (IQR) | 42 (35-50) | 45 (36-52) | 0.400 |
|  |  |  |  |
| *First radiological assessment* |  |  |  |
| Median size (IQR) | 41 (37-48) | 45 (37-57) | 0.062 |
|  |  |  |  |
| *Final (PD) assessment* |  |  |  |
| Median size (IQR) | 43 (37-46) | 44 (37-56) | 0.445 |

*Abbreviations.* ctDNA, circulating tumour DNA; IQR, interquartile range; N, number; PD, progressive disease.

**Supplementary Table 3. Association of plasma *AR* copy number and metastatic pattern in mCRPC**

|  | ***AR* normal** | ***AR* gain** |  |
| --- | --- | --- | --- |
|  | **N (%)** | **N (%)** | **p** |
| **Bone metastases** |  |  |  |
| *Baseline* |  |  |  |
| *Bone extent* |  |  |  |
| Oligometastatic (≤5 lesions) (O) | 27 (51.9) | 7 (28.0) |  |
| Polymetastatic (between 6 and 19 lesions) (D) | 10 (19.2) | 7 (28.0) |  |
| Widespread (≥20 lesions) (S) | 15 (28.9) | 11 (44.0) | 0.140 |
|  |  |  |  |
| Presence of pathological tissue | 3 (2.0) | 4 (5.5) | 0.224 |
| Presence of pathological fracture | 4 (2.7) | 1 (1.4) | 0.527 |
|  |  |  |  |
| *First radiological assessment* |  |  |  |
| *Bone extent* |  |  |  |
| Oligometastatic (≤5 lesions) (O) | 20 (37.0) | 4 (17.4) |  |
| Polymetastatic (between 6 and 19 lesions) (D) | 17 (31.5) | 8 (34.8) |  |
| Widespread (≥20 lesions) (S) | 17 (31.5) | 11 (47.8) | 0.150 |
|  |  |  |  |
| Presence of pathological tissue | 0 | 0 | - |
| Presence of pathological fracture | 3 (2.0) | 1 (1.4) | 0.726 |
|  |  |  |  |
| *Final (PD) assessment* |  |  |  |
| *Bone extent* |  |  |  |
| Oligometastatic (≤5 lesions) (O) | 13 (26.0) | 4 (18.2) |  |
| Polymetastatic (between 6 and 19 lesions) (D) | 16 (32) | 6 (27.3) |  |
| Widespread (≥20 lesions) (S) | 21 (42.0) | 12 (54.5) | 0.555 |
|  |  |  |  |
| Presence of pathological tissue | 7 (4.8) | 2 (2.7) | 0.721 |
| Presence of pathological fracture | 3 (2.0) | 1 (1.4) | 0.726 |
|  |  |  |  |
| **Lymph nodal metastases** |  |  |  |
| *Baseline* |  |  |  |
| Lymph nodal site |  |  |  |
| Thoracic (T) | 1 (4.5) | 0 |  |
| Abdominal (A) | 17 (77.3) | 6 (66.7) |  |
| Both (TA) | 4 (18.2) | 3 (33.3) | 0.561 |
|  |  |  |  |
| Number of lymph node metastases |  |  |  |
| <5 | 11 (50.0) | 5 (55.6) |  |
| ≥5 | 11 (50.0) | 4 (44.4) | 0.779 |
| Max size (cm) median value (IQR) | 22 (17-38) | 19 (15-23) | 0.570 |
|  |  |  |  |
| *First radiological assessment* |  |  |  |
| Lymph nodal site |  |  |  |
| Thoracic (T) | 1 (6.3) | 0 |  |
| Abdominal (A) | 11 (68.7) | 3 (60.0) |  |
| Both (TA) | 4 (25.0) | 2 (40.0) | 0.720 |
|  |  |  |  |
| Number of lymph node metastases |  |  |  |
| <5 | 10 (62.5) | 5 (100) |  |
| ≥5 | 6 (37.5) | 0 | 0.262 |
| Max size (cm) median value (IQR) | 26 (13-34) | 15 (14-19) | 0.017 |
|  |  |  |  |
| *Final (PD) assessment* |  |  |  |
| Lymph nodal site |  |  |  |
| Thoracic (T) | 2 (7.7) | 0 |  |
| Abdominal (A) | 20 (76.9) | 6 (66.7) |  |
| Both (TA) | 4 (15.4) | 3 (33.3) | 0.394 |
|  |  |  |  |
| Number of lymph node metastases |  |  |  |
| <5 | 13 (50.0) | 7 (77.8) |  |
| ≥5 | 13 (50.0) | 2 (22.2) | 0.147 |
| Max size (cm) median value (IQR) | 27 (20-41) | 18 (15-22) | 0.011 |

*Abbreviation.* AR, androgen receptor, IQR, interquartile range; N, number; PD, progressive disease.

**Supplementary Table 4. Association of plasma *AR* copy number and prostatic involvement in mCRPC**

|  | ***AR* normal** | ***AR* gain** |  |
| --- | --- | --- | --- |
|  | **N (%)** | **N (%)** | **p** |
| **Primary tumour** |  |  |  |
| *Baseline* |  |  |  |
| Median size (IQR) | 43 (35-53) | 42 (38-47) | 0.812 |
|  |  |  |  |
| *First radiological assessment* |  |  |  |
| Median size (IQR) | 42 (41-53) | 42 (37-47) | 1.000 |
|  |  |  |  |
| *Final (PD) assessment* |  |  |  |
| Median size (IQR) | 44 (38-56) | 39 (35-46) | 0.289 |

*Abbreviations.* ctDNA, circulating tumour DNA; IQR, interquartile range; N, number; PD, progressive disease.

**Supplementary Table 5. Association of ctDNA fraction and metastatic pattern at baseline and 3-month therapy**

|  | **ctDNA - baseline** | **ctDNA - 3 months** |
| --- | --- | --- |
| **Metastatic pattern** | **Median value (IQR)** | **Median value (IQR)** |
| Oligometastatic (≤5 lesions) (O) | 0.12 (0.10-0.20) | 0.16 (0.11-0.24) |
| Polymetastatic (between 6 and 19 lesions) (D) | 0.17 (0.12-0.29) | 0.33 (0.17-0.54) |
| Widespread (≥20 lesions) (S) | 0.38 (0.28-0.64) | 0.46 (0.26-0.58) |
| **p-value** | 0.0003 | 0.0003 |

*Abbreviations.* ctDNA, circulating tumour DNA; IQR, interquartile range; N, number; PD, progressive disease.

**Supplementary Table 6.** **Univariate analysis of PFS and OS as a function of ctDNA change (from baseline to 3-month therapy)**

|  | **PFS** | | **OS** | |
| --- | --- | --- | --- | --- |
| **ctDNA change (from baseline to 3-month therapy)** | **HR (95% CI)** | **p** | **HR (95% CI)** | **p** |
| **No^*^** | 1.00 |  | 1.00 |  |
| **Yes^**^** | 2.06 (1.30-3.27) | 0.002 | 1.82 (1.15-2.88) | 0.010 |

^*^ High/high and low/low based on median ctDNA value

^**^ Low/high (excluding only 4 high/low cases)

*Abbreviations.* CI, confidence interval; ctDNA, circulating tumour DNA; HR, hazard ratio; OS, overall survival; PFS, progression-free survival.

**Supplementary Table 7. Univariate analysis of PFS and OS**

|  | **PFS** | | **OS** | |
| --- | --- | --- | --- | --- |
|  | **HR (95% CI)** | **p** | **HR (95% CI)** | **p** |
| **Age** (≥74 vs <74 years) | 1.03 (0.79-1.35) | 0.803 | 0.98 (0.74-1.29) | 0.881 |
| **Bone metastasis** (yes vs no) | 1.35 (0.91-2.01) | 0.135 | 1.60 (1.05-2.45) | 0.030 |
| **Metastatic pattern variation** |  |  |  |  |
| D 🡪 D / D 🡪 S (vs O 🡪 O) | 1.28 (0.64-2.53) | 0.483 | 2.19 (1.06-4.51) | 0.034 |
| S 🡪 S (vs O 🡪 O) | 1.43 (0.78-2.63) | 0.251 | 2.40 (1.25-4.60) | 0.008 |
| O 🡪 D / O 🡪 S (vs O 🡪 O) | 2.06 (0.98-4.34) | 0.056 | 2.22 (1.04-4.72) | 0.039 |
| **Lymph nodes metastasis** (yes vs no) | 1.01 (0.76-1.32) | 0.978 | 0.92 (0.70-1.22) | 0.586 |
| **Visceral metastasis** (yes vs no) | 1.87 (1.30-2.68) | 0.0007 | 2.47 (1.71-3.58) | <0.0001 |
| **Presence of pain** (yes vs no) | 1.03 (0.71-1.51) | 0.859 | 0.92 (0.62-1.37) | 0.678 |
| **ECOG PS** (2 vs 0-1) | 3.26 (1.91-5.57) | <0.0001 | 5.88 (3.39-10.22) | <0.0001 |
| **Gleason score** (≥8 vs 6-7) | 1.10 (0.83-1.47) | 0.513 | 1.05 (0.78-1.41) | 0.765 |
| **Prior docetaxel** (yes vs no) | 2.28 (1.44-3.63) | 0.0005 | 1.93 (1.22-3.07) | 0.0005 |
| **ctDNA fraction** (> vs < median value) | 2.41 (1.71-3.38) | <0.0001 | 2.20 (1.55-3.12) | <0.0001 |
| **% ctDNA variation** |  |  |  |  |
| Low-High (vs Low-Low) | 2.11 (1.13-3.92) | 0.018 | 1.77 (0.94-3.32) | 0.076 |
| High-High (vs Low-Low) | 3.32 (1.92-5.74) | <0.0001 | 2.37 (1.39-4.02) | 0.001 |
| **PSA value** (> vs < median value) | 1.34 (0.86-2.09) | 0.194 | 1.59 (1.01-2.49) | 0.045 |

*Abbreviations.* CI, confidence interval; ctDNA, circulating tumour DNA; D, polymetastatic disease (between 6 and 19 lesions); ECOG, Eastern Cooperative Oncology Group; HR, hazard ratio; OS, overall survival; PFS, progression-free survival; PS, performance status, PSA, prostate specific antigen; S, widespread disease (≥20 lesions).

**Supplementary Figure 1.** Variation in ctDNA fraction from baseline to 3-month therapy


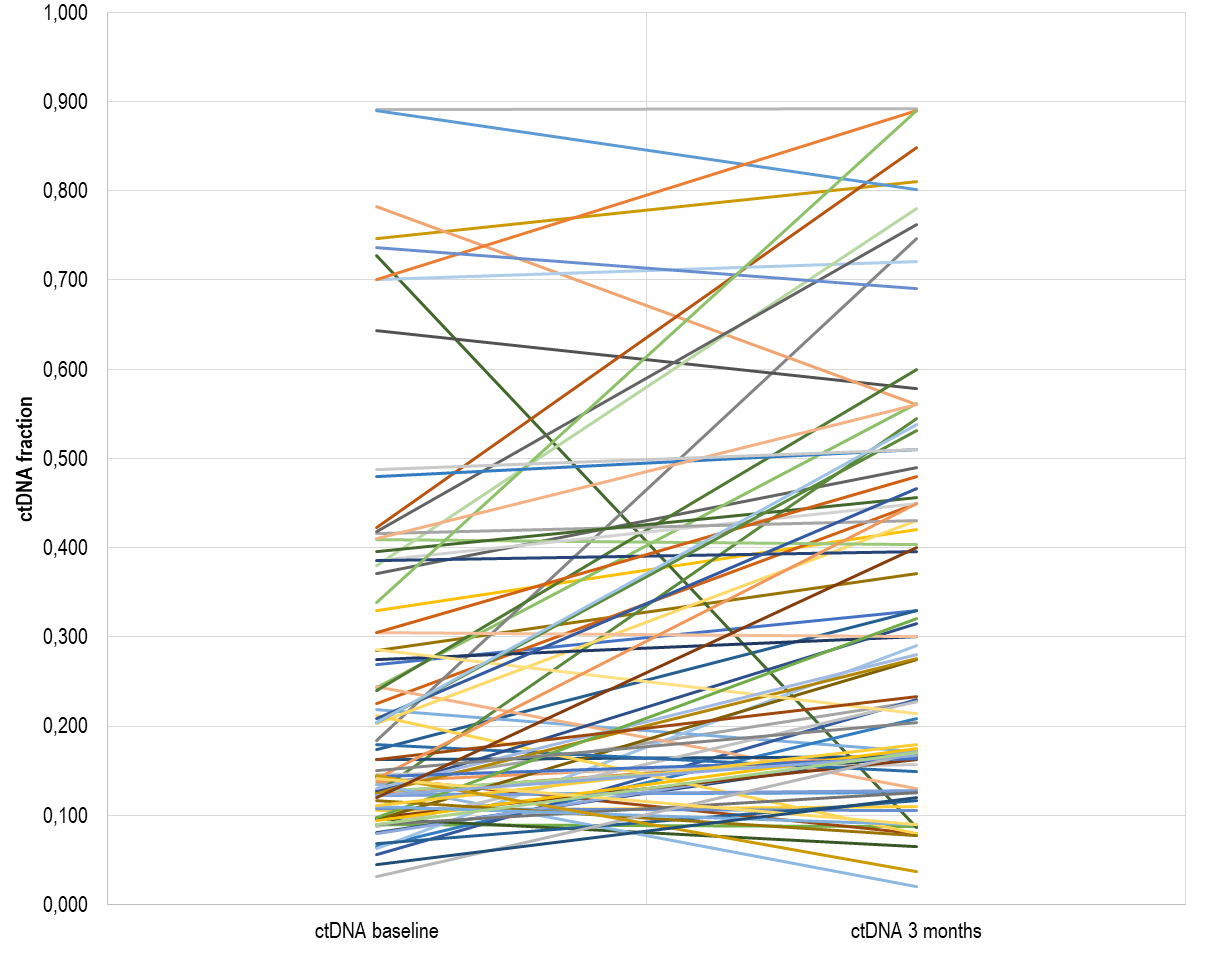

Supplement: oyaf107_suppl_Supplementary_Tables_1-7_Figures_1 [file oyaf107_suppl_supplementary_tables_1-7_figures_1.docx]
